# From the Service of Prof. N. S. Davis, in the Medical Wards of Mercy Hospital, February 18, 1869

**Published:** 1869-04

**Authors:** W. A. Barstow


### FROM THE SERVICE OF PROF. N. S. DAVIS, IN THE MEDICAL WARDS OF MERCY HOSPITAL, February 18, 1869.

Reported by W. A. BARSTOW.

Gentlemen:—This man tells us that, some six months ago, while engaged in sinking a shaft in the vicinity of Morris, in this State, he was attacked with pain in the anterior portion of the thigh, stopping short above the knee. The pain, however, soon extended to the iliac and lumbar regions, and subsequently across to the epigastrium, which has continued since the last of July, with but little relief. He has been under treatment for rheumatism. I first saw him at my office one or two weeks since. I find, on examining the spine at the lower, or next to the lower, lumbar vertebra, there is a little prominence. There is but little tenderness over the spinous processes, but directly along the right side of the vertebra, for several inches, it is extremely sensitive, and seems to be almost exclusively limited to the right side. There seems to be a very little swelling, but which may be due to dry cups which were applied two days since. He will allow any degree of flexion of his thigh, but if you extend the leg and carry it back of its fellow, the pain is reflected along the margin of crest of the ilium and abdomen. If he sits down, or stoops to pick up anything, instead of bending forward as a well person would, he squats down, endeavoring to keep his spine as straight as possible.

This symptom is of great value in forming a diagnosis, and raises the question as to what the disease is, and what has produced it? To the experienced observer, his symptoms immediately suggest one of the three following diseases:—1. Inflammation of the psoas muscle, or adjacent areolar tissue, tending to the formation of an abscess. 2. Disease of the vertebra. 3. Inflammation of the right half of the spinal cord. Any one of these three diseases would be brought to mind in the investigation. First, we will take up affections involving the psoas muscle. Pain in the front part of the thigh and abdomen would first lead us to suspect that this muscle was involved. The first effect of inflammation in contact with a muscle is to render it rigid. The tendency, if the psoas muscle is inflamed, is to relax the abdomen by flexing the thigh on the pelvis. If he puts his leg down straight, or stands square on his feet, he will lean his body forward, so as to still relax the muscle on the anterior part of the spine; and, if in bed, he will be found to have the thigh drawn up. The pain accompanying psoas abscess is dull and deep-seated, extending from the abdomen to the junction of the lumbar vertebra, and across by the crest of the ilium. The most diagnostic signs being, pain down the thigh, and in one side of the abdomen, and the flexion of the thigh. If asked to flex the thigh strongly, he cannot, the pain being much increased. If you examine the patient while on his back, you will generally find, on pressure, a degree of tumefaction along the inside of the anterior part of the crest of the ilium, or a sense of fulness and tenderness, which does not correspond to the opposite side. In the present case, none of these symptoms are present, except the pain in the anterior part of the thigh, and pain in extending the leg backward. Is the disease in the vertebra? One symptom exactly corresponds with the early stage of spinal disease, *viz.*:—the mode of stooping down, which may be noticed in children who have spinal disease, coming on even before they complain much. Instead of stooping over, they will likewise squat; and if they can reach anything to support them in rising, they will do so. This symptom, however, is not restricted to disease of the bones of the spine, but may be present in any affection that renders the spine sore.

Six months have elapsed, and there is no perceptible alteration, except in the one spinous process previously mentioned; hence, we are not justified in saying that he has disease of the vertebra from the manner of stooping alone.

The tenderness along the sides of the vertebræ is severe, while in disease of the bone there is rarely any muscular soreness or neuralgic pains, until the disease has progressed to such an extent as to make some degree of deformity perceptible; then you have severe paroxysms of pain in the epigastric region, in the intercostal spaces, or horizontally around the abdomen. There is seldom any evidence, in the early stage, of interference with muscular action. But in this case, pain in the muscles was one of the first symptoms; and, taking this together with the facts that it is of six months' duration, and no deformity; while the pain follows certain nerves, with acute and severe tenderness along right side of the spine; increased by exercise, and occasional cramps in the abdominal muscles; all of which point to the right half of the spinal cord, corresponding with the lower half of the dorsal vertebra, as the seat of disease.

Both sets of nerves are involved—namely, those of sensation and motion. From a close investigation of his case, my opinion is, that he has chronic inflammation of the membranes of the spinal cord, along the lower third of the dorsal vertebræ. If that is the case, has it produced any disorganization of structure? We answer no, or it would have left him with paralysis; and, if there was effusion, this would have certainly produced paralysis in some degree. If it be simple chronic inflammation, involving the roots of nerves, what is the appropriate treatment? We answer, dry cupping, followed by belladonna plasters or hypodermic injections of atropine; and, internally, we will first put him on the following treatment:—

℞. Tinct. Cimmicifuga, ---------------------- ℥ij.

Tinct. Stramonii, --------------------------- ℥ss.

Iodide Potassa, ---------------------------- ʒiiss.

Simple Syrup, ------------------------------- ℥iss.

Mix.

Of which, we will give a teaspoonful every four hours; and, three times a day, a powder, consisting of—

℞. Potassa Nitras, } āā, -------------------- gr. viij.

Pulv. Doveri, }

Hyd. Chlor. Mite, ---------------------- gr. ij.

The calomel to be discontinued as soon as its alterative effects are perceptible in the breath or gums.

These means, with rest in the horizontal position, will be likely to remove the disease in from four to six months.

Common tumblers form good cups for broad surfaces like the back, as was shown by their application in this case before the Class.

---

Gentlemen:—The next case to which I propose to call your attention to-day is one of partial hemiplegia.

The patient is a carpenter, and says that, for two or three months before the attack, he was at times light-headed and dizzy, and was afraid to venture upon the scaffolding, and consequently went to work in the shop.

It seems that, the night of the attack, the patient was out later than usual with some friends, and during the evening had indulged in a glass or two of stimulants, but not enough to feel the effects to such an extent that he did not know all that occurred.

After parting with his friends, he started for home. Before he proceeded far, however, he says that he was gradually taken blind and dizzy, and finally fell on the sidewalk, and became partially unconscious. When he recovered himself, he found he had no power in his right arm nor leg, but succeeded in dragging himself to a doorway.

Until within the past week, he has been under treatment at Madison, Wis., where he was living at the time of the attack. At present, you would hardly know that his leg was affected in walking; but, on closer observation, you would notice that he raised it with difficulty when attempting to step over anything. His face has improved equally with his leg, but his arm is still nearly useless. Can shut his hand quite tight, and has a good degree of power in the flexor muscles; but the action of the extensors is much impaired, and supination is also rendered imperfect. This shows us that the whole set of extensors, from he shoulder down, are more feeble than the flexors.

The first item we wish to investigate is the seat of the disease. The paralysis of the arm and leg are but symptoms, and may arise from three sources:—1. The muscles themselves, as in lead palsy. 2. The spinal cord. 3. The brain itself. The symptoms of giddiness and the paralysis, extending to the face, must necessarily involve the nerves within the cranium; hence, we refer it to the brain. If there had been no paralysis of the face and tongue, we might have presumed it was in the spinal canal. The patient's mind being clear, while there is giddiness and dimness of vision at times, we refer the seat of disease to the base and central portions of the brain.

In determining the nature of a disease like this, it is essential to get as accurate a history of the case as possible.

First, we may have paralysis come on suddenly, with severe pain, as in the case of the patient up-stairs, to which your attention was a few days since directed, indicating a pathological change of an apoplectic character.

In this case, it has come on gradually, and became fully developed when under the effects of stimulants. Rest improves his muscular power, while exercise uniformly exhausts it.

This would indicate the existence of some gradual change at the base of the brain, like syphilitic thickening of the dura mater, the growth of a tumor, or gradual softening of brain substance.

We will endeavor to draw the line of distinction between these pathological conditions.

The symptoms of white atrophy or softening do not correspond to those of the case before us. That disease comes on insidiously, by simple impairment of the muscular power, the legs or arms requiring an extra exertion on the part of the patient to use them. It is a gradual weakening, which, when once begun, continues on worse and worse, and involves loss of coördinating power and strength, without complete paralysis, until the last stage of disease.

Now this man was not progressively losing the strength and coördinate power of his muscles, but merely giddiness and headache. And, instead of steady increase of his disease, under treatment he has been decidedly improving, and now only complains of paralysis of the right arm, with pain in the shoulder of the affected side, and occasional darting of pain up the back part of the head.

It seems that, several years ago, this patient contracted syphilis; and he now has maculæ on the skin, and what he calls catarrhal disease in the nostrils. And it is highly probable that his giddiness and paralysis arise from thickening of the dura mater over the sphenoid bone.

It is of the utmost importance to ascertain the cause, in such cases as this, in order to know how to treat the disease intelligibly. He has been taking iodide of potassium and strychnia; and I think, had the iodide been combined with minute doses of the bichloride of mercury, it would have benefited him still more. The question is, had the disease involved the bones—either the ethmoid and palate, or any portion of the sphenoid? If it has, the prognosis would be considered unfavorable, although not terminating for three or four years. If it is confined to the soft parts, we would expect a recovery.

Viewing the case in this light, we will put him on the following treatment, with an occasional intermission of a week:—

℞. Iodide Potassium, ʒiij.
Bichlor. Hydr., gr. j.
Syr. and Aquæ, ℥iv.
M.

Of which, we will give a teaspoonful four times a day. We will also use a weak solution of carbolic acid as an injection, which may be applied by means of a curved syringe through the posterior nares. We may derive some benefit from muscular tonics when the muscles are flaccid; but strychnia and electricity should never be used to any extent, if there is rigidity of the muscular fibres.

Under the foregoing treatment, we may expect the patient to recover a good degree of health, and the use of his arm, in from four to six weeks. It will depend, however, upon whether the bones at the base of the brain are affected or not.

